# Prognostic factors for lymph node infestation, long-term survival and recurrence rates in patients with vaginal cancer: a population-based study in Germany

**DOI:** 10.1007/s00432-026-06436-6

**Published:** 2026-03-11

**Authors:** Shirin Wenning, Michael Gerken, Thomas Papathemelis, Olaf Ortmann, Bianca Franke, Ian Wittenberg, Christina Walter, Constanze Schneider, Andrea Sackmann, Sylke Ruth Zeissig, Fabian Reinwald, Natalie Rath, Kerstin Weitmann, Jacqueline Müller-Nordhorn, Caroline Herr, Monika Klinkhammer-Schalke, Simone Marnitz, Elisabeth Christine Sturm-Inwald

**Affiliations:** 1 Tumour Center, Center for Quality Management and Health Services Research, University of Regensburg, Regensburg, Germany; 2 Bavarian Cancer Registry - Regional Center Regensburg, Bavarian Health and Food Safety Authority (LGL), Regensburg, Germany; 3 Department of Gynecology and Obstetrics, St. Marien Hospital, Amberg, Germany; 4 Department of Gynecology and Obstetrics, University Medical Center Regensburg, Regensburg, Germany; 5 Association of German Tumor Centers ADT, Berlin, Germany; 6 Saxony-Anhalt Cancer Registry, Magdeburg, Germany; 7 Southwest German Tumor Center CCC Tübingen University Hospital, Tübingen, Germany; 8 Clinical-Epidemiological Cancer Registry Brandenburg-Berlin gGmbH, Berlin, Germany; 9 Hessian Cancer Registry, Frankfurt am Main, Germany; 10 Bavarian Cancer Registry, Regional Center Würzburg, Würzburg, Germany; 11 Cancer Registry of Rhineland-Palatinate in the Institute for Digital Health Data RLP, Mainz, Germany; 12 Saarland Cancer Registry, Saarbrücken, Germany; 13 Mecklenburg-Western Pomerania Cancer Registry, Greifswald, Germany; 14 Bavarian Cancer Registry, Bavarian Health and Food Safety Authority, Nuremberg, Germany; 15 Bavarian Health and Food Safety Authority, Environmental Health, Munich, Germany; 16 Institute and Clinic for Occupational, Social and Environmental Medicine, University Hospital, LMU Munich, Munich, Germany; 17 Pettenkofer School of Public Health, Munich, Germany; 18 RadioOnkologie at Vosspalais, Private surgery, Berlin, Germany; 19 Institute of Clinical Epidemiology and Biometry (ICE-B), University of Würzburg, Würzburg, Germany; 20 Department of Epidemiology and Preventive Medicine, University of Regensburg, Franz-Josef-Strauß-Allee 11, 93053 Regensburg, Germany; 21 Bavarian Cancer Registry, Bavarian Health and Food Safety Authority, Regensburg, Germany

**Keywords:** Vaginal cancer, Risk factors, Survival analysis, Recurrence rates, Lymph nodes, Treatment outcomes, Cancer registry

## Abstract

**Purpose:**

Vaginal cancer (ICD-10: C52) is one of the rarest gynecological malignancies, with limited research on its frequency, long-term survival, therapy-dependent survival, and follow-up of recurrences. This knowledge deficit may lead to potential consequences such as medical over-, under- or wrong therapy.

**Methods:**

This nationwide retrospective population-based registry study analyzed patients with vaginal cancer diagnosed between 2000 and 2022 in Germany. A total cohort of 1325 patients was included. To assess prognostic factors related to lymph node involvement, overall survival, recurrence rates, and therapy-dependent survival were analyzed using binary logistic regression, the Kaplan-Meier method, and univariable and multivariable Cox regression.

**Results:**

Mean age at diagnosis was 68.5 years and median age was 70.4 years. 30.0% (*n* = 398) of patients showed lymph node involvement. Younger age at diagnosis (< 60 years), larger tumor size, and lymphatic invasion were noted as significant risk factors for lymph node involvement in multivariable analysis. The 5-year overall survival rate was 53.8% (95%-CI 50.9–56.7), significantly influenced by age at diagnosis, nodal status, and tumor size in multivariable analysis. Cumulative recurrence rates for locoregional and distant metastases were 20.4% and 14.3% after 5 years, increasing to 24.3% and 16.5% after 10 years. The most common primary treatment was surgery (39.2%, *n* = 454). Surgery plus radiochemotherapy (OP + RCT) provided the most favorable outcome as a primary treatment, whereas radiotherapy alone showed the least benefit of all treatment options.

**Conclusion:**

Our study identifies important prognostic factors influencing vaginal cancer survival and offers information on treatment optimization.

**Supplementary Information:**

The online version contains supplementary material available at 10.1007/s00432-026-06436-6.

## Introduction

Vaginal cancer is one of the rarest gynecological malignancies. In the European Union, a disease is considered rare if it affects no more than 5 in 10,000 people (Bundesgesundheitsministerium 2025). In 2022, the global estimated incidence was 18,800 cases, representing 0.1% of all cancers (Bray et al. 2024). In Germany, approximately 500 new cases are expected annually (Schnürch et al. 2019). In 2020, the German Robert Koch Institute (RKI) documented 390 new cases and 164 deaths (RKI und GEKID, 2023).

Vaginal neoplasms occur predominantly in women over 70 years of age. One of the most significant risk factors is the infection with HPV, detected in 74% of malignant vaginal tumors. A prevention can be achieved through vaccination (Schnürch et al. 2019). The most frequent histological type is squamous cell carcinoma (SCC), accounting for 85–87% of cases, followed by adenocarcinoma in 10% of patients (Albuquerque et al. 2022). Sarcomas and melanomas are rare (< 7%) (Dittmer et al. 2011). SCC is more prevalent in elderly women, with individuals over 70 years accounting for more than half of all cases. In contrast, vaginal adenocarcinoma particularly clear cell carcinoma associated with intrauterine exposure to diethylstilbestrol (DES) – predominantly occurs in younger women (Stein et al. 2021; Kaltenecker et al. 2025).

The leading symptom is vaginal bleeding, often accompanied by signs of local infiltration, such as frequent urination (Chow et al. 2021). Local relapses are relatively frequent, presenting in 23–26% of patients. In 90% of cases, relapses occur within the first 5 years after primary diagnosis (Jhingran 2022). Therefore, follow-up physical examination is of great significance. In Germany, continued medical follow-up is recommended every three months in the first three years, followed by semi-annual examinations in the next two years (Schnürch et al. 2019). Subsequently, an annual gynecological examination should be carried out (Schnürch et al. 2019). In addition, the stage at initial diagnosis significantly impacts the chances of survival. Hence, patients with localized stage have better survival rates compared to patients with more advanced stages (Adams et al. 2021).

Currently, there are only a few studies available addressing vaginal cancer. Few studies provide information on prognostic factors and survival outcomes. There is little clinical registry data available which makes it difficult to conduct high-level studies with real-world data. Moreover, the guideline recommendations are also not clearly defined yet, resulting in analogous treatment of vaginal and vulvar carcinoma, making evidence-based patient care more difficult. In 2020, a U.S. study utilizing the Surveillance, Epidemiology, and End Results (SEER) database was conducted to estimate survival rates and identify prognostic factors. However, recurrence rates were not analyzed (Huang et al. 2020). Similarly, a German study, published in 2022, presented epidemiological registry data, without providing information on recurrence rates or treatment details (Forner 2023). This draws attention to the existing research gap in the areas of therapy and recurrences.

Thus, the goal of the present study is to evaluate prognostic factors for lymph-node involvement, long-term survival and recurrence rates based on clinical cancer registry data from Germany. Furthermore, it aims to analyze data on therapy-dependent survival to optimize future treatments.

## Materials and methods

### Study design and patient cohort

Our nationwide population-based, retrospective cohort study used data from 15 German clinical cancer registries from the Federal States of Baden-Württemberg, Bavaria, Berlin, Brandenburg, Hesse, Lower Saxony, Mecklenburg-Western Pomerania, Saxony, Saxony-Anhalt and Thuringia. A pooled anonymized dataset was analyzed. In total, 1325 patients were included, who were diagnosed with an invasive vaginal neoplasm (ICD-10 C52) between January 2000 and December 2022. The collected data, as well as the retrospective analyses of the patients were anonymized and consistent and in accordance with the state cancer registry laws of the participating registries.

### Patient inclusion and exclusion criteria

**Fig. 1:**
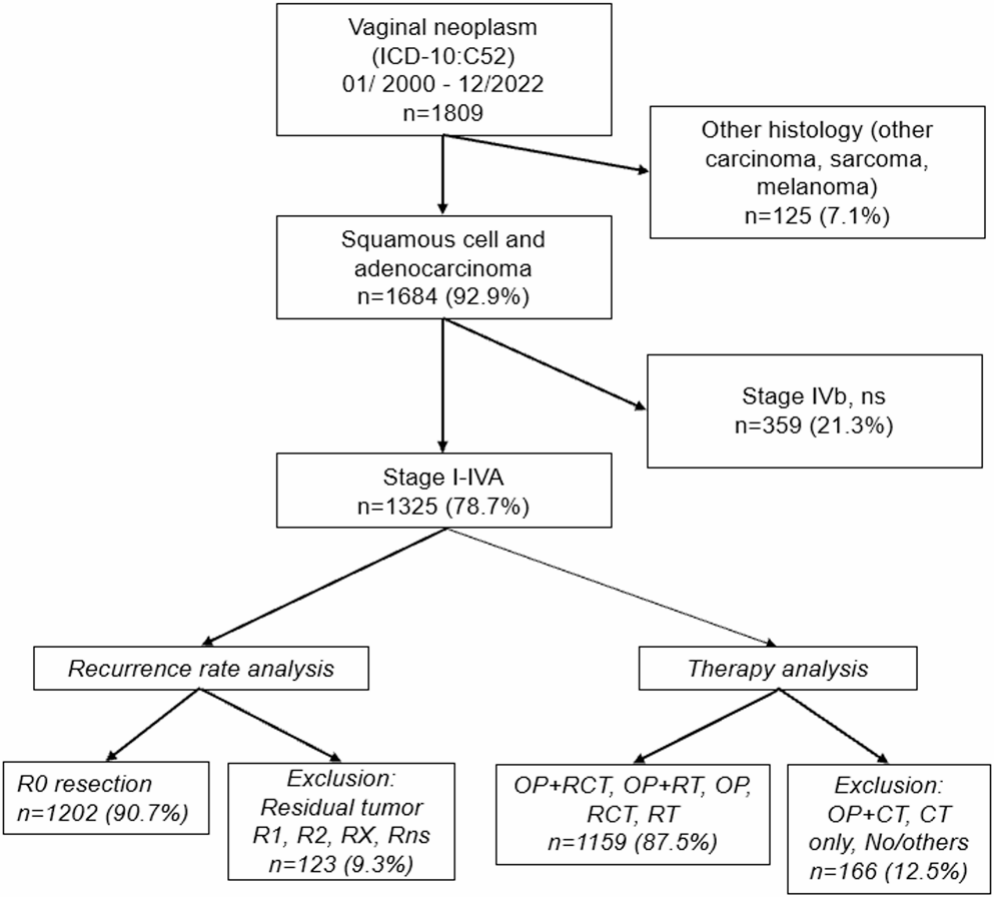
Flowchart showing inclusion and exclusion criteria

Inclusion and exclusion criteria are depicted in Fig. 1. Patients were classified according to the UICC (Union international contre le cancer) TNM staging to assess the extent of lymph node involvement. Only patients with a histology of SCC and adenocarcinoma were included in the study, whereas rare histological subtypes and patients with primary distant metastases (UICC stage IVB) were excluded to focus on tumor recurrence. Recurrences occurring within the first three months after diagnosis were not counted as recurrences. To achieve sufficient case numbers, gradings G1 and G2, as well as G3 and G4 were combined. Any primary lymph node involvement was marked as N+.

Data on lymphatic and venous invasion were incomplete due to registry-specific reporting requirements that were not mandatory throughout the study period. Analyses of these real- life variables were therefore not restricted to cases with complete data, in order to reflect the real-life data refraining from exclusion of cases, and the results were interpreted as exploratory. As the missing data are unlikely to be random, no imputation methods were applied.

Furthermore, only patients with R0 resection and those treated with the major therapy forms surgery alone, surgery combined with radiotherapy or chemoradiotherapy, radiotherapy alone or chemoradiotherapy—were involved in analyses of recurrence rates, with each therapy form including an adequate patient number of more than 100 patients.

Some potentially relevant prognostic factors, such as tumor localization within the vagina, HPV status, performance status, comorbidities, chemotherapy protocols, and radiation dosage/fractionation, were not consistently reported across all registries and years. Therefore, these variables could not be included in the multivariable analyses.

### Statistical analysis

Means and medians with 95%-confidence intervals (95%-CI) as well as interquartile range (IQR), minimum and maximum values were used to characterize continuous data. Categorial data were displayed as absolute frequencies and relative percentages. Continuous data were evaluated with the two-tailed Student’s t-test in case of normal distribution, otherwise the Mann-Whitney U test was used. For assessing the independence between categorical variables, the Pearson’s Chi-square test was utilized. Multivariable binary logistic regression was conducted to analyze the likelihood of lymph node involvement, reported as odds ratios (OR) along with the 95%-CI.

Overall survival (OS) rates were estimated from information given by clinical reports, death certificates, and registration offices. Relapses were categorized into locoregional recurrences, comprising relapse of the primary and/or regional lymph nodes, and distant metastases. The date of primary diagnosis until the first event determined the survival times in years for OS and time to first recurrence. The cut-off date was determined as 31 December 2022. Mean and median follow-up was estimated by applying the reverse Kaplan-Meier method. The Kaplan-Meier method was used to estimate the endpoints 5-year-OS rate, the recurrence-free survival rate, and the cumulative recurrence rate. The two-sided log-rank test (i.e., Mantel- Cox) was used to present the statistical significance in survival outcomes. The statistical significance threshold was defined as 0.05. To determine the effect of the patient and tumor characteristics on survival, the Cox proportional hazard models with univariable and multivariable regression analyses were applied. In addition, sensitivity analyses were carried out with a backward stepwise selection with a cut-off value of 0.10. For covariates, such as age at diagnosis, histological type, grading, UICC staging, tumor size, nodal status, lymph vessel, and venous invasion, the Hazard ratio (HR) for overall survival and cumulative recurrence was estimated. The evaluation of HRs along with the 95%-CI were conducted. They were deemed statistically significant, if the 95%-CI did not include 1.0 and the two-sided p values were less than 0.05.

To check the assumption of proportionality, Kaplan-Meier curves were used and interaction terms linked to OS time were included in the Cox-regression model. IBM^®^ SPSS^®^ Statistics (IBM Corp. IBM SPSS for Windows, Version 29.0. Armonk, NY, USA) was applied to analyze all data.

## Results

### Patient characteristics

The cohort study included information on 1325 patients obtained from German clinical tumor registries. An overview of the tumor and patient characteristics is summarized in Table 1. The mean age at diagnosis was 68.5 years (95%-CI 67.8–69.3, SD 13.7) and the median age was 70.4 years (95%-CI 69.4–71.3, IQR 59.0-79.2). Minimum and maximum values were 23.5 and 99.7 years. The interquartile range was 59.0-79.2 years. 27.0% (*n* = 358) of patients were under 60 years of age, while 22.4% (*n* = 297) were between 60 and 69 years old. The majority of our patients, 29.2% (*n* = 387), were aged 70–79, and a significant proportion of 21.4% (*n* = 283) were over 79 years old. The leading histological type was squamous cell carcinoma (93.0%, *n* = 1232). The predominant stage according to UICC was stage I (37.4%, *n* = 495). Correspondingly, T1 (42.6%, *n* = 564) was the most frequently detected tumor size. In most patients G1/G2 (55.8%, *n* = 740) grading was found. 30.0% (*n* = 398) of patients had lymph node involvement (N+). Although limited data was available on lymph vessel and venous vessel invasion, the existing information indicated that only few patients did show any involvement (L1 = 14.4%, n _L1_ =191, V1 = 3.8%, n _V1_ =50).

**Table 1:** Patient and tumor characteristics

		Diagnosis ICD-10	
		C52 vagina	
		n	%
Age at diagnosis (years)	0–59	358	27.0
	60–69	297	22.4
	70–79	387	29.2
	80+	283	21.4
Age at diagnosis (metric)	*Mean (SD)*	*68.5*	*(13.7)*
	*Median (IQR)*	*70.4*	*(59.0–79.2)*
Histological type	SCC	1232	93.0
	Adenocarcinoma	93	7.0
Stage UICC	I	495	37.4
	II	266	20.1
	III	359	27.1
	IVA	205	15.5
Tumor size (T)	T1	564	42.6
	T2	408	30.8
	T3	150	11.3
	T4	203	15.3
Nodal status (N)	N0	927	70.0
	N+	398	30.0
Grading (G)	G1/2	740	55.8
	G3/4	477	36.0
	GX/ns	108	8.2
Lymph vessel invasion (L)	L0	388	29.3
	L1	191	14.4
	LX/ns	746	56.3
Venous invasion (V)	V0	480	36.2
	V1	50	3.8
	VX/ns	795	60.0
	Total	1325	100.0

IQR=Interquartile range, SD= Standard deviation, SCC= Squamous cell carcinoma, ns = non-specified

### Risk factors for lymph node involvement

Lymph node involvement (N+) was found in 398 patients (30.0%). Mean and median age at diagnosis for patients with lymph node infestation was 68.2 years and 70.0 years. Among those patients, the majority had a histology of squamous cell carcinoma (92.0%, *n* = 366) (Suppl. Tab. A). Significant risk factors for nodal involvement at primary diagnosis were younger age at diagnosis (< 60 years) with significantly lower OR of 0.644–0.648 for older age groups, larger tumor size compared to T1 tumors (T2: OR 3.2, 95%- CI 2.3–4.5, *p* < 0.001; T3: OR 6.1, 95%-CI 4.0–9.5, *p* < 0.001; T4: OR 8.1, 95%-CI 5.5–12.1, *p* < 0.001) and lymphatic invasion L1 vs. L0 (OR: 5.1, 95%-CI 3.1–8.4, *p* < 0.001), derived from multivariable binary logistic regression. Histological type, grading and venous invasion were not found to be independent risk factors (Suppl. Tab. B).

### Overall survival analyses

**Fig. 2:**
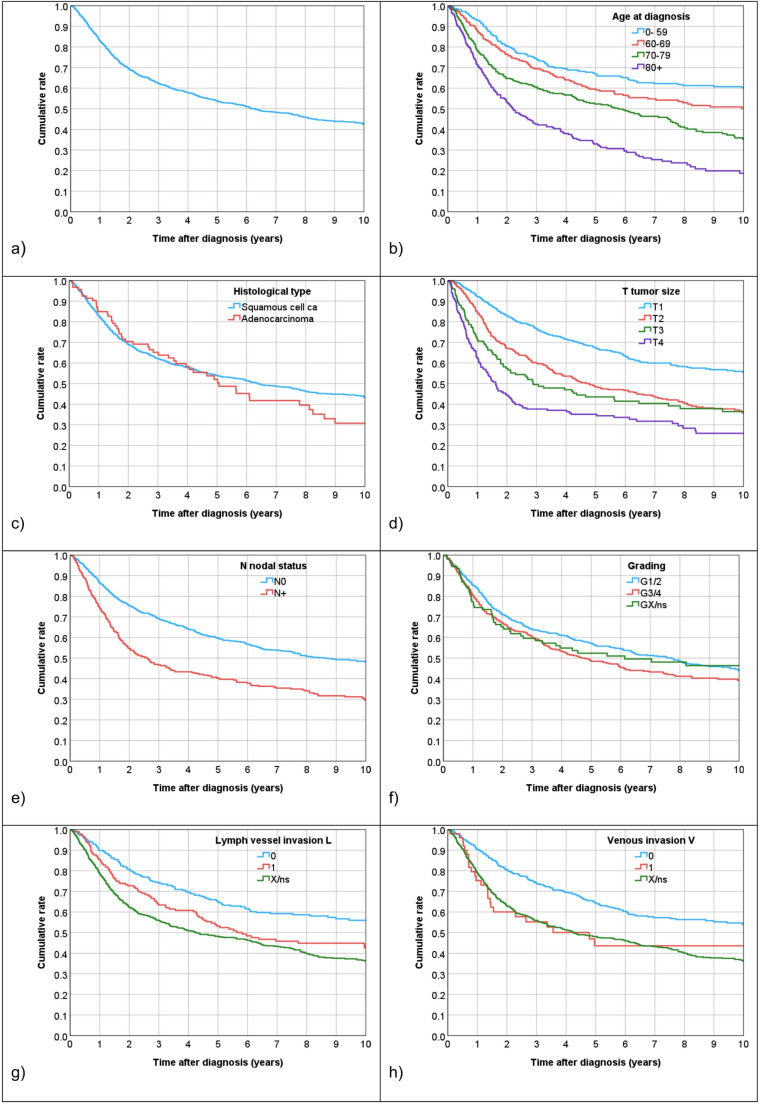
Overall survival according to patient and tumor characteristics (Kaplan-Meier). ns = non-specified

Mean and median follow-up time was 9.3 years (95%-CI 8.9–9.8) and 8.2 years (95%- CI 7.6–8.9). During the follow-up period, 684 of the 1325 patients died (48.4%). The 5-year survival rate was 53.8% (95%-CI 50.9–56.7). The median survival time was 6.3 years (95%-CI 5.2–7.3). The overall survival curves in the entire cohort and according to the patient and tumor characteristics are shown in Fig. 2. Results from univariable and multivariable Cox-regressions are listed in Table 2.

**Table 2:** Univariable and multivariable Cox regression for overall survival according to risk factors

		Univariable Cox-regression					Multivariable Cox-regression			
				95%-CI					95%-CI	
		*p*	HR	Lower		Upper	*p*	HR	Lower	Upper
Age at diagnosis (years)	< 60	< 0.001	1.000				< 0.001*	1.000		
	60–69	0.003	1.437	1.131		1.826	0.004	1.419	1.115	1.805
	70–79	< 0.001	2.127	1.711		2.644	< 0.001	2.222	1.784	2.767
	80+	< 0.001	3.231	2.580		4.047	< 0.001	2.979	2.370	3.744
Histological type	SCC		1.000					1.000		
	Adenocarcinoma	0.639	1.071	0.803		1.429	0.225	1.197	0.895	1.601
Tumor size (T)	T1	< 0.001	1.000				< 0.001*	1.000		
	T2	< 0.001	1.758	1.463		2.112	< 0.001	1.545	1.273	1.874
	T3	< 0.001	2.017	1.577		2.581	< 0.001	1.607	1.238	2.087
	T4	< 0.001	2.856	2.303		3.540	< 0.001	2.322	1.822	2.958
Nodal status (N)	N0		1.000					1.000		
	N+	< 0.001	1.704	1.458		1.991	0.003	1.307	1.098	1.556
Grading (G)	G1/2	0.125	1.000				0.131*	1.000		
	G3/4	0.042	1.180	1.006		1.384	0.073	1.160	0.986	1.364
	GX/ns	0.537	1.092	0.826		1.443	0.617	0.931	0.702	1.234
Lymph vessel invasion (L)	L0	< 0.001	1.000				0.937*	1.000		
	L1	0.012	1.390	1.075		1.798	0.813	0.965	0.716	1.300
	LX/ns	< 0.001	1.779	1.476		2.145	0.921	1.022	0.666	1.568
Venous invasion (V)	V0	< 0.001	1.000				0.152*	1.000		
	V1	0.008	1.721	1.149		2.577	0.060	1.514	0.982	2.333
	VX/ns	< 0.001	1.706	1.439		2.022	0.329	1.222	0.817	1.828

* p-value in line of reference denotes p-value for entire variable, CI=Confidence interval, HR=Hazard ratio, SCC=Squamous cell carcinoma, ns = non-specified

In the univariable Cox-regression analysis all variables except histological type and grading showed a significant influence on OS. The risk factors remaining significant in the multivariable analysis were age at diagnosis, nodal status, and tumor size. Patients aged over 70 and over 80 years demonstrated a highly significant decrease in OS with an HR of 2.2 (95%- CI 1.8–2.8, *p* < 0.001) and 3.0 (95%-CI 2.4–3.7, *p* < 0.001) compared to younger patients. Additionally, OS significantly worsened with tumor extension to T2, T3 and T4, with an HR of 1.5 (95%-CI 1.3–1.9, *p* < 0.001), 1.6 (95%-CI 1.2–2.1, *p* < 0.001) and 2.3 (95%- CI 1.8–3.0, *p* < 0.001), compared to T1. Patients with involved lymph nodes (N+) showed a highly significant worse OS with an HR of 1.3 (95%-CI 1.3–1.1, *p* < 0.001) in contrast to patients with nodal status N0.

G3/G4 grading displayed a statistical significant influence in univariable analysis with an HR of 1.2 (95%-CI 1.0–1.4, *p* = 0.042) in contrast to G1/G2. However, multivariable analysis revealed a borderline non-significant effect despite an HR of 1.2 (95%-CI 1.0–1.4, *p* = 0.073).

In the multivariable analysis within the full model, venous spread showed a marginally non-significant effect in patients with V1 compared to V0 involvement, but had an HR of 1.5 (95%- CI 1.0–2.3, *p* = 0.060). After performing a stepwise backward selection, the *p*-value for venous invasion was 0.032 for V1 compared to V0.

### Cumulative recurrence rate

After excluding patients who were not R0, 1202 patients remained in the cohort. Of these, 197 (16.4%) had locoregional relapse and 130 (10.8%) had distant metastases. After 5 years, the cumulative locoregional recurrence rate was 20.4% (95%-CI 17.6–23.2), increasing slightly to 24.3% (95%-CI 21.1–27.6) after 10 years (Supp. Fig. A). As to distant metastases, the 5-year and 10-year recurrence rates were 14.3% (95%-CI 11.8–16.8) and 16.5% (95%-CI 13.7–19.3). The most common distant metastases were found in the lung (3.3%, *n* = 40), followed by bones (2.3%, *n* = 28) and liver (2.1%, *n* = 25) (Suppl. Tab. C). None of the tumor characteristics demonstrated a significant effect on locoregional recurrences neither in univariable nor in multivariable analysis (Suppl. Tab. D).

However, several factors proved to be significant for the occurrence of distant metastases (Suppl. Tab. E). Patients with adenocarcinoma had a significantly higher recurrence rate with an HR of 2.6 (95%-CI 1.6–4.2, *p* < 0.001) in comparison to SCC. Tumor size T4 also showed a highly significant impact on recurrence rate with an HR of 2.8 (95%- CI 1.7–4.8, *p* < 0.001) as opposed to T1. In contrast to patients with venous invasion V0, patients with V1 posed an independent risk of distant metastasis (HR = 2.3, 95%- CI 1.1–5.0, *p* = 0.034).

### Therapy

The final analysis included all patients, also those with resection statuses other than R0. Of the initial 1325 patients, 166 were excluded because they had no record of one of the primary therapies previously described. This resulted in a final cohort of 1159 patients. Only treatment groups with more than 100 patients were included, while groups with fewer than 100 patients were excluded from the analysis. In this cohort, the median follow-up time was 8.2 years (95%- CI 7.6–9.0). The most common primary treatment was surgery (OP, *n* = 454, 39.2%), followed by a combination of surgery plus radiotherapy (OP + RT, *n* = 236, 20.4%).

Definitive radiotherapy (RT) was used in 196 patients (16.9%), whereas 147 patients (12.7%) had radiochemotherapy (RCT). The least common treatment was surgery plus radiochemotherapy (OP + RCT, *n* = 126, 10.9%, Table 4). Patient and tumor characteristics according to therapy groups are depicted in Table 3.

**Table 3:** Patient and tumor characteristics according to therapy group in the total cohort

		Primary therapy											
		OP + RCT		OP + RT		OP		RCT		RT		Total	
		n	%	n	%	n	%	n	%	n	%	n	%
Age at diagnosis (years)	0–49	29	23.0	35	14.8	52	11.5	16	10.9	5	2.6	137	11.8
	50–59	33	26.2	37	15.7	77	17.0	33	22.4	11	5.6	191	16.5
	60–69	34	27.0	61	25.8	118	26.0	37	25.2	17	8.7	267	23.0
	70–79	26	20.6	73	30.9	136	30.0	46	31.3	59	30.1	340	29.3
	80+	4	3.2	30	12.7	71	15.6	15	10.2	104	53.1	224	19.3
Histological type	SCC	115	91.3	204	86.4	428	94.3	143	97.3	188	95.9	1078	93.0
	Adenocarcinoma	11	8.7	32	13.6	26	5.7	4	2.7	8	4.1	81	7.0
Stage UICC	I	25	19.8	125	53.0	275	60.6	7	4.8	38	19.4	470	40.6
	II	30	23.8	40	16.9	79	17.4	31	21.1	49	25.0	229	19.8
	III	51	40.5	56	23.7	65	14.3	67	45.6	67	34.2	306	26.4
	IVA	20	15.9	15	6.4	35	7.7	42	28.6	42	21.4	154	13.3
Tumor size (T)	T1	38	30.2	143	60.6	295	65.0	12	8.2	44	22.4	532	45.9
	T2	54	42.9	68	28.8	102	22.5	53	36.1	78	39.8	355	30.6
	T3	15	11.9	10	4.2	23	5.1	40	27.2	32	16.3	120	10.4
	T4	19	15.1	15	6.4	34	7.5	42	28.6	42	21.4	152	13.1
Nodal status (N)	N0	67	53.2	177	75.0	394	86.8	73	49.7	117	59.7	828	71.4
	N+	59	46.8	59	25.0	60	13.2	74	50.3	79	40.3	331	28.6
Grading (G)	G1	4	3.2	15	6.4	31	6.8	4	2.7	6	3.1	60	5.2
	G2	70	55.6	118	50.0	233	51.3	74	50.3	102	52.0	597	51.5
	G3/4	48	38.1	83	35.2	173	38.1	46	31.3	68	34.7	418	36.1
	GX/ns	4	3.2	20	8.5	17	3.7	23	15.6	20	10.2	84	7.2
Lymph vessel invasion (L)	L0	29	23.0	103	43.6	240	52.9	2	1.4	1	0.5	375	32.4
	L1	41	32.5	51	21.6	77	17.0	3	2.0	5	2.6	177	15.3
	LX/ns	56	44.4	82	34.7	137	30.2	142	96.6	190	96.9	607	52.4
Venous invasion (V)	V0	48	38.1	122	51.7	291	64.1	1	0.7	1	0.5	463	39.9
	V1	15	11.9	12	5.1	17	3.7	1	0.7	0	0.0	45	3.9
	VX/ns	63	50.0	102	43.2	146	32.2	145	98.6	195	99.5	651	56.2
	Total	126	100.0	236	100.0	454	100.0	147	100.0	196	100.0	1159	100.0

OP + RCT=Operation+Radiochemotherapy, OP + RT=Operation+Radiotherapy, OP=Operation, RCT=Radiochemotherapy, RT=Radiotherapy, SCC=Squamous cell carcinoma, ns = non-specified

**Table 4:** Results from multivariable Cox regression for overall survival according to therapy in total cohort, and in subgroups by age, tumor size T and nodal status N

				Multivariable Cox-regression			
						95%-CI	
Subgroup	Primary therapy	n	%	p*	HR	Lower	Upper
Total	OP + RCT	126	10.9	0.008	1.000		
	OP + RT	236	20.4	0.129	1.302	0.926	1.830
	OP	454	39.2	0.184	1.253	0.898	1.747
	RCT	147	12.7	0.928	1.018	0.687	1.509
	RT	196	16.9	0.004	1.711	1.184	2.472
Age 0–59	OP + RCT	62	18.9	0.553	1.000		
	OP + RT	72	22.0	0.177	1.526	0.826	2.818
	OP	129	39.3	0.419	1.280	0.703	2.329
	RCT	49	14.9	0.834	1.078	0.533	2.179
	RT	16	4.9	0.210	1.821	0.714	4.646
Age 60–69	OP + RCT	34	12.7	0.287	1.000		
	OP + RT	61	22.8	0.465	1.289	0.653	2.543
	OP	118	44.2	0.969	0.986	0.502	1.939
	RCT	37	13.9	0.981	0.991	0.450	2.183
	RT	17	6.4	0.100	2.004	0.875	4.588
Age 70–79	OP + RCT	26	7.6	0.047	1.000		
	OP + RT	73	21.5	0.169	1.654	0.808	3.387
	OP	136	40.0	0.080	1.880	0.928	3.810
	RCT	46	13.5	0.326	1.500	0.668	3.368
	RT	59	17.4	0.011	2.652	1.256	5.599
Age 80+	OP + RCT	4	1.8	0.343	1.000		
	OP + RT	30	13.4	0.500	0.663	0.201	2.189
	OP	71	31.7	0.725	0.821	0.273	2.463
	RCT	15	6.7	0.159	0.378	0.098	1.462
	RT	104	46.4	0.873	0.918	0.322	2.618
T1	OP + RCT	38	7.1	0.580	1.000		
	OP + RT	143	26.9	0.875	1.053	0.554	2.003
	OP	295	55.5	0.612	0.849	0.451	1.599
	RCT	12	2.3	0.320	0.516	0.140	1.900
	RT	44	8.3	0.982	0.991	0.459	2.139
T2	OP + RCT	54	15.2	0.054	1.000		
	OP + RT	68	19.2	0.568	1.161	0.695	1.941
	OP	102	28.7	0.571	1.158	0.698	1.919
	RCT	53	14.9	0.934	0.974	0.517	1.831
	RT	78	22.0	0.022	1.938	1.102	3.409
T3	OP + RCT	15	12.5	0.556	1.000		
	OP + RT	10	8.3	0.675	1.373	0.313	6.020
	OP	23	19.2	0.824	1.186	0.263	5.340
	RCT	40	33.3	0.865	1.120	0.303	4.136
	RT	32	26.7	0.283	2.085	0.545	7.986
T4	OP + RCT	19	12.5	0.052	1.000		
	OP + RT	15	9.9	0.600	1.331	0.457	3.872
	OP	34	22.4	0.059	2.441	0.968	6.159
	RCT	42	27.6	0.796	1.123	0.466	2.705
	RT	42	27.6	0.068	2.399	0.937	6.143
N0	OP + RCT	67	8.1	0.225	1.000		
	OP + RT	177	21.4	0.346	1.248	0.787	1.981
	OP	394	47.6	0.561	1.142	0.731	1.784
	RCT	73	8.8	0.935	0.976	0.547	1.742
	RT	117	14.1	0.086	1.551	0.940	2.559
N+	OP + RCT	59	17.8	0.005	1.000		
	OP + RT	59	17.8	0.443	1.236	0.720	2.122
	OP	60	18.1	0.071	1.634	0.958	2.785
	RCT	74	22.4	0.954	0.984	0.561	1.724
	RT	79	23.9	0.006	2.174	1.253	3.771

*p-value in line of reference denotes p-value for entire variable. CI=Confidence interval, HR= Hazard ratio, OP + RCT=Operation+Radiochemotherapy, OP + RT=Operation+Radiotherapy, OP=Operation, RCT=Radiochemotherapy, RT=Radiotherapy, SCC=Squamous cell carcinoma, ns = non-specified

OP, either as a single or combination treatment, was predominantly performed on patients under 50 years of age (84.7%, *n* = 116). As to RT, more than half of the patients (53.1%, *n* = 104) were older than 80 years, whereas only 16.8% (*n* = 33) were younger than 70 years. Among patients receiving OP alone, 60.6% (*n* = 275) were diagnosed with UICC stage I, while 7.7% (*n* = 35) were at stage IVA. This suggests that the use of definitive OP decreases with increasing UICC stage. Furthermore, among those who underwent OP, 86.8% (*n* = 394) showed no lymph node involvement (N0), 52.9% (*n* = 240) had no lymph vessel invasion (L0) and 64.1% (*n* = 291) were free of venous invasion (V0). In contrast, among patients receiving combination therapy (OP + RCT), a higher proportion presented with more advanced tumor stages. 46.8% (*n* = 59) showed lymph node involvement, 32.5% (*n* = 41) had lymph vessel invasion and 11.9% (*n* = 15) displayed venous invasion. Similarly, among patients treated with RCT and definitive RT, 50.3% (*n* = 74) and 40.3% (*n* = 79) of patients had infested lymph nodes.

In addition, a multivariable Cox-regression was performed to analyze OS according to therapy within the total cohort, as well as in subgroups categorized by age, tumor size T, and nodal status N (Table 4). OP + RCT was the reference group. Definitive RT was the only therapy that had a significantly worse influence on OS with an HR of 1.7 (95%- CI 1.2–2.5, *p* = 0.004) in comparison to OP + RCT. With the exception of the 70–79 age group, where RT alone shortened OS (HR 2.7, 95%-CI 1.3–5.6, *p* = 0.011) compared to OP + RCT, none of the other treatment options displayed a statistically significant influence on OS across the other age groups. Additionally, definitive RT rendered a worse OS compared to OP + RCT in T2 tumors (HR = 1.9, *p* = 0.022, 95%-CI 1.1–3.4) and in patients with lymph node involvement (HR = 2.2, *p* = 0.006, 95%-CI 1.3–3.8). In patients with lymph node involvement, RCT demonstrated the best OS (HR 0.9, *p* = 0.954, 95%-CI 0.6–1.7) and also the second-best OS rate overall compared to OP + RCT. It appears that monotherapy consistently showed worse OS compared to combination therapies.

However, this observation appears to be influenced by baseline differences and should not be interpreted as evidence of treatment superiority. Summarizing the results, the best OS was found in patients with OP + RCT, followed by RCT. Definitive RT provided the least benefit compared to the other treatment options discussed. The observed findings are likely driven by differences in patient selection and tumor characteristics, not differences in treatment efficacy, and should thus be interpreted with caution.

## Discussion

Vaginal cancer is a rare malignancy. To date, research based on retrospective and prospective studies remains limited. Since the data on vaginal cancer therapy is based on limited retrospective clinical reports, the German S2k-Level guidelines often refer to management recommendations analogously to vulvar or cervix carcinoma (Schnürch et al. 2019).

In Germany, (Forner 2023) presented stratified survival data from a large cohort of vaginal cancer patients using data from German epidemiological registries. Our study expands the data by including information on recurrence rates and treatment modalities using data from population-based clinical cancer registries.

Our research demonstrates that younger age at diagnosis, larger tumor size, and lymphatic invasion are the most important prognostic factors for lymph node involvement. OS was significantly lower for patients with older age at diagnosis, larger tumor size and lymph node involvement. Recurrence rates continue to rise after 5 years. Recurrence rates for distant metastases were negatively influenced by the histological type of adenocarcinoma, T4 tumors, and venous invasion. As to treatment options, patients with radiochemotherapy and surgery plus radiochemotherapy had the best survival benefit. Patients with definitive radiotherapy showed the worst OS.

It is important to note, that treatment comparisons are subject to confounding by indication, as older and more frail patients with advanced disease were more likely to receive definitive radiotherapy. Despite multivariable adjustment, residual confounding cannot be excluded. In addition, definitive radiotherapy was associated with poorer overall survival, likely reflecting advanced age, disease stage, and patient selection rather than treatment inefficacy.

According to the guidelines, the diagnostic workup of vaginal carcinoma should include clinical examinations, followed by imaging studies and biopsies for histological analyses. For large tumors, screening for distant metastases should be carried out prior to initiating treatment. Treatment approaches are indicated based on UICC TNM classification. Early-stage tumors (T1 N0 M0) were primarily treated with surgery, while in more advanced tumors (T2 N0 M0, T3 N0 M0, T1-3 N1 M0) radio(chemo) therapy is the preferred treatment. The decision to combine surgery with radiotherapy or chemotherapy for stages T1-T4 (any N M0) depends on the individual case, especially for more invasive cancers. Follow-up examinations are recommended every quarter in the first three years after diagnosis, followed by semi-annual check-ups from years four to five, and annual examinations thereafter (Schnürch et al. 2019).

In our study, the median age at diagnosis of 70.4 years was consistent with the median age of 70 years presented by Forner (2023). Wu et al. (2008) presented a slightly younger age of 68 years, while Prameela et al. (2016) reported an even younger median age of 64.28 years. In addition to the prognostic factors for lymph node involvement identified in this study, Baral et al. (2022) pointed out that that the location of the tumor is a significant factor. In particular, lesions involving the posterior wall of the vagina had a higher incidence of lymph node metastases.

In our study, the 5-year OS rate was 53.8%, which differed from the results of other German studies. Forner (2023) reported a lower rate of 48.6%, and Buttmann-Schweiger et al. (2019) reported a rate of 44%. In contrast, Meixner et al. (2023) observed a higher 5-year-OS rate of 62.8%. The comparability of the 5-year OS rate in our study to other studies is limited due to the exclusion of patients with primary distant metastases.

Similar to our results, Huang et al. (2020) pointed out that the case-specific survival rate decreases with increasing age. Lymph node involvement has a considerable influence on OS. This result was also found by Forner (2023), who showed a median OS of 24 months in case of lymph node involvement compared to median OS of 83 months in patients without lymph node involvement.

Lymph node metastases are commonly associated with SCC and distant metastases with adenocarcinoma (Yang et al. 2020). This explains the significant impact of adenocarcinoma on the recurrence rates of distant metastases found in this study. Additionally, recurrence rates for both locoregional and distant metastases worsen slightly after 5–10 years, which raises the question of whether the follow-up period should be extended.

In terms of therapy, most studies recommend radiotherapy as the primary treatment method for advanced tumors (Adams et al. 2021; Jhingran 2022), while our study shows its limitations. Our analysis compares the OS of different treatment options with the OS of OP plus RCT.

In the multivariable Cox-regression definitive radiotherapy shows a significantly worse OS. However, this finding may be explained by the advanced age of patients who received RT: 53.1% were older than 80 years. Interestingly, the adverse effect on OS persists after adjustment in the multivariable analysis. Not only radiotherapy, but all other treatment options also show a worse OS than surgery plus RCT.

An American study by Rajagopalan et al. published in 2014, highlighted the significant impact of concomitant radiochemotherapy over definitive radiotherapy, showing better survival outcomes: the 5-year OS rate was improved by 6.9% (Rajagopalan et al. 2014). The chemotherapeutic agents most commonly used were cisplatin and 5-fluorouracil (Kulkarni et al. 2022).

### Strengths and limitations

This is the first study analyzing clinical cancer registry data on vaginal carcinoma in Germany. This study has several strengths, including a large cohort of 1325 patients from 15 German cancer registries and a long follow-up time of 22 years, allowing for comprehensive analysis of long-term survival, recurrence patterns, and therapy-dependent outcomes. However, it has its limitations, especially its retrospective design which may result in selection and information bias. A further limitation of our study is the absence of certain prognostic factors, including tumor location, HPV status, patient performance status, comorbidities, chemotherapy protocols, and radiation dose/fractionation. These factors were not consistently documented in the registries and could influence survival and recurrence outcomes. Consequently, unmeasured confounding cannot be fully excluded. In addition this study is based on German cancer registry data and reflects national treatment and follow-up procedures. While generalization to other countries should be done with caution, major prognostic factors such as age, tumor size and nodal status are likely relevant beyond the German healthcare system.

## Conclusion

The presented data from the population-based and therefore representative clinical cancer registries, which are based on a large cohort and a 22-year follow-up period, provide important information for the future treatment and follow-up of vaginal cancer patients in Germany. Given the rarity of vaginal cancer and the limited research available, our study significantly contributes to the existing knowledge. In particular, the information on therapy-dependent survival will improve the understanding of treatment outcomes in Germany. This will allow more specialized treatment of vaginal cancer, rather than treating it analogously to cervix or vulva carcinoma. Prognostic factors for lymph node involvement included age at diagnosis (< 60 years), larger tumor size and lymph vessel invasion. Independent prognostic factors associated with poorer OS included older age at diagnosis, positive nodal status and larger tumor size. The fact that recurrence rates continue to rise after 5 years, underlines the importance of long-term follow-up and raises the question of whether follow-up frequency should be increased after 5 years. Surgery combined with RCT provided the best treatment outcomes compared to other therapies. The recurring link between monotherapy and shorter overall survival, along with the less favorable outcomes observed with definitive radiotherapy in certain subgroups, could serve as a basis for generating new hypotheses. These observations highlight the importance of conducting prospective studies to identify optimal treatment strategies and to more precisely determine which patient subgroups may benefit from specific therapeutic approaches. Therefore personalized treatment approaches based on patient characteristics (age, tumor size, lymph node involvement) are needed to guarantee the best patient care. Our study has highlighted important prognostic factors and treatment options, contributing to a better understanding of vaginal cancer and improved patient care.

## Supplementary Information

Below is the link to the electronic supplementary material.

Supplementary Material 1

## Data Availability

The datasets generated and/or analyzed during the current study are not publicly available because of the data security of the cancer registries.
