# Supplementary material for: Prognostic factors for lymph node infestation, long-term survival and recurrence rates in patients with vaginal cancer: a population-based study in Germany
Source: J Cancer Res Clin Oncol. 2026 Mar 11;152(3):63. doi: 10.1007/s00432-026-06436-6 (PMC12979751; doi:10.1007/s00432-026-06436-6)
Supplement: Supplementary file 1 — Supplementary Material 1 [file 432_2026_6436_MOESM1_ESM.docx]

**Supplements**

**Suppl. Tab. A: Patient and tumor characteristics by lymph node infestation**

|  | | Nodal status (N) | | | | | |  |
| --- | --- | --- | --- | --- | --- | --- | --- | --- |
|  |  | N0 | | N+ | | Total | | *X^2^* |
|  |  | n | % | n | % | n | % | *p* |
| Age at diagnosis (years) | 0- 59 | 238 | 25.7% | 120 | 30.2% | 358 | 27.0% | 0.202 |
|  | 60-69 | 216 | 23.3% | 81 | 20.4% | 297 | 22.4% |  |
|  | 70-79 | 280 | 30.2% | 107 | 26.9% | 387 | 29.2% |  |
|  | 80+ | 193 | 20.8% | 90 | 22.6% | 283 | 21.4% |  |
| Age at diagnosis (metric) | *Mean (SD)*  *Median (IQR)* | *68.6*  *70.5* | *(13.4)*  *(59-79)* | *68.2*  *70.0* | *(13.4)*  *(59-79)* | *68.5*  *70.4* | *(13.7)*  *(59-79)* |  |
| Histological type | SCC | 866 | 93.4% | 366 | 92.0% | 1232 | 93.0% | 0.340 |
|  | Adenocarcinoma | 61 | 6.6% | 32 | 8.0% | 93 | 7.0% |  |
| Tumor size (T) | T1 | 496 | 53.5% | 68 | 17.1% | 564 | 42.6% | <0.001 |
|  | T2 | 267 | 28.8% | 141 | 35.4% | 408 | 30.8% |  |
|  | T3 | 76 | 8.2% | 74 | 18.6% | 150 | 11.3% |  |
|  | T4 | 88 | 9.5% | 115 | 28.9% | 203 | 15.3% |  |
| Grading (G) | G1/2 | 539 | 58.1% | 201 | 50.5% | 740 | 55.8% | 0.034 |
|  | G3/4 | 318 | 34.3% | 159 | 39.9% | 477 | 36.0% |  |
|  | GX/ns | 70 | 7.6% | 38 | 9.5% | 108 | 8.2% |  |
| Lymph vessel invasion (L) | L0 | 344 | 37.1% | 44 | 11.1% | 388 | 29.3% | <0.001 |
|  | L1 | 111 | 12.0% | 80 | 20.1% | 191 | 14.4% |  |
|  | LX/ns | 472 | 50.9% | 274 | 68.8% | 746 | 56.3% |  |
| Venous invasion (V) | V0 | 390 | 42.1% | 90 | 22.6% | 480 | 36.2% | <0.001 |
|  | V1 | 31 | 3.3% | 19 | 4.8% | 50 | 3.8% |  |
|  | VX/ns | 506 | 54.6% | 289 | 72.6% | 795 | 60.0% |  |
|  | Total | 927 | 100.0% | 398 | 100.0% | 1325 | 100.0% |  |

IQR=Interquartile range

SD= Standard deviation

SCC= Squamous cell carcinoma

ns=non-specified

**Suppl. Table A:** Patient and tumor characteristics by lymph node infestation

**Supp Tab. B: Results from multivariable binary logistic regression: odds ratios for the risk of lymph node infestation depending on patient and tumor characteristics**

|  |  | Multivariable binary logistic regression | | | |
| --- | --- | --- | --- | --- | --- |
|  |  |  |  | 95%-CI | |
|  |  | *p* | OR | lower | upper |
| Age at diagnosis (years) | < 60 | 0.036* | 1.000 |  |  |
|  | 60-69 | 0.022 | 0.644 | 0.442 | 0.939 |
|  | 70-79 | 0.015 | 0.648 | 0.457 | 0.918 |
|  | 80+ | 0.022 | 0.644 | 0.442 | 0.938 |
| Histological type | SCC |  | 1.000 |  |  |
|  | Adenocarcinoma | 0.275 | 1.319 | 0.802 | 2.168 |
| Tumor size (T) | T1 | <0.001* | 1.000 |  |  |
|  | T2 | <0.001 | 3.181 | 2.265 | 4.467 |
|  | T3 | <0.001 | 6.138 | 3.982 | 9.461 |
|  | T4 | <0.001 | 8.128 | 5.460 | 12.099 |
| Grading (G) | G1/2 | 0.235* | 1.000 |  |  |
|  | G3/4 | 0.099 | 1.265 | 0.957 | 1.672 |
|  | GX/ns | 0.977 | 0.993 | 0.620 | 1.591 |
| Lymph vessel invasion (L) | L0 | <0.001* | 1.000 |  |  |
|  | L1 | <0.001 | 5.060 | 3.059 | 8.370 |
|  | LX/ns | <0.001 | 4.101 | 1.878 | 8.958 |
| Venous invasion (V) | L0 | 0.461* | 1.000 |  |  |
|  | L1 | 0.426 | 0.741 | 0.355 | 1.548 |
|  | LX/ns | 0.252 | 0.660 | 0.324 | 1.344 |

*p-value in line of reference denotes p-value for entire variable

CI=Confidence interval

OR=Odds ratio

SCC= Squamous cell carcinoma

ns=non-specified

**Suppl. Table B**: Results from multivariable binary logistic regression: odds ratios for the risk of lymph node infestation depending on patient and tumor characteristics

**Suppl. Tab. C: Distribution and Localization of distant metastases recurrences**

|  | | n | % |
| --- | --- | --- | --- |
| Distant metastases recurrence | Yes | 130 | 10,8% |
|  | No | 1072 | 89,2% |
| Brain | Yes | 3 | 0,2% |
|  | No | 1199 | 99,8% |
| Lung | Yes | 40 | 3,3% |
|  | No | 1162 | 96,7% |
| Pleura | Yes | 2 | 0,2% |
|  | No | 1200 | 99,8% |
| Liver | Yes | 25 | 2,1% |
|  | No | 1177 | 97,9% |
| Peritoneal | Yes | 13 | 1,1% |
|  | No | 1189 | 98,9% |
| Adrenal | Yes | 1 | 0,1% |
|  | No | 1201 | 99,9% |
| Bone | Yes | 28 | 2,3% |
|  | No | 1174 | 97,7% |
| Lymph nodes | Yes | 22 | 1,8% |
|  | No | 1180 | 98,2% |
| Skin | Yes | 8 | 0,7% |
|  | No | 1194 | 99,3% |
| Others/ns | Yes | 24 | 2,0% |
|  | No | 1178 | 98,0% |

ns=non-specified

**Suppl. Table C:** Distribution and Localization of distant metastases recurrences

**Suppl. Tab. D: Cumulative locoregional recurrence rates according to patient and tumor characteristics (univariable and multivariable Cox regression)**

|  |  | Univariable Cox-regression | | | | | Multivariable Cox-regression | | | |
| --- | --- | --- | --- | --- | --- | --- | --- | --- | --- | --- |
|  |  |  |  | 95%-CI | | |  |  | 95%-CI | |
|  |  | *p* | HR | lower | | upper | *p* | HR | lower | upper |
| Age at diagnosis (years) | < 60 | 0.082 | 1.000 | |  |  | 0.091 | 1.000* |  |  |
|  | 60-69 | 0.117 | 1.337 | 0.930 | | 1.922 | 0.096 | 1.363 | 0.947 | 1.963 |
|  | 70-79 | 0.312 | 1.208 | 0.838 | | 1.744 | 0.182 | 1.287 | 0.889 | 1.863 |
|  | 80+ | 0.236 | 0.746 | 0.459 | | 1.212 | 0.377 | 0.801 | 0.489 | 1.310 |
| Histological type | SCC |  | 1.000 |  | |  |  | 1.000 |  |  |
|  | Adenocarcinoma | 0.371 | 1.255 | 0.763 | | 2.064 | 0.547 | 1.166 | 0.707 | 1.925 |
| Tumor size (T) | T1 | 0.618 | 1.000 |  | |  | 0.339 | 1.000* |  |  |
|  | T2 | 0.363 | 1.161 | 0.842 | | 1.600 | 0.202 | 1.249 | 0.888 | 1.757 |
|  | T3 | 0.828 | 0.945 | 0.569 | | 1.570 | 0.820 | 1.064 | 0.622 | 1.821 |
|  | T4 | 0.302 | 1.260 | 0.812 | | 1.953 | 0.102 | 1.488 | 0.924 | 2.396 |
| Nodal status (N) | N0 |  | 1.000 |  | |  |  | 1.000 |  |  |
|  | N+ | 0.225 | 1.208 | 0.890 | | 1.639 | 0.179 | 1.264 | 0.898 | 1.778 |
| Grading (G) | G1/2 | 0.083 | 1.000 |  | |  | 0.099 | 1.000* |  |  |
|  | G3/4 | 0.199 | 0.818 | 0.603 | | 1.111 | 0.133 | 0.788 | 0.578 | 1.075 |
|  | GX/ns | 0.045 | 0.518 | 0.272 | | 0.987 | 0.081 | 0.560 | 0.292 | 1.075 |
| Lymph vessel invasion (L) | L0 | 0.045 | 1.000 |  | |  | 0.157 | 1.000* |  |  |
|  | L1 | 0.639 | 0.905 | 0.595 | | 1.375 | 0.248 | 0.751 | 0.461 | 1.221 |
|  | LX/ns | 0.015 | 0.684 | 0.504 | | 0.929 | 0.056 | 0.497 | 0.242 | 1.019 |
| Venous invasion (V) | V0 | 0.103 | 1.000 |  | |  | 0.702 | 1.000* |  |  |
|  | V1 | 0.755 | 1.130 | 0.523 | | 2.441 | 0.583 | 1.254 | 0.559 | 2.813 |
|  | VX/ns | 0.045 | 0.747 | 0.561 | | 0.993 | 0.465 | 1.283 | 0.657 | 2.506 |

*p-value in line of reference denotes p-value for entire variable

CI=Confidence interval

HR=Hazard ratio

SCC=Squamous cell carcinoma

ns=non-specified

**Suppl. Table D:** Cumulative locoregional recurrence rates according to patient and tumor characteristics (univariable and multivariable Cox regression)

**Suppl. Tab. E: Cumulative distant metastasis recurrence rates according to patient and tumor characteristics (univariable and multivariable Cox regression)**

|  |  | Univariable Cox-regression | | | | | Multivariable Cox-regression | | | |
| --- | --- | --- | --- | --- | --- | --- | --- | --- | --- | --- |
|  |  |  |  | 95%-CI | | |  |  | 95%-CI | |
|  |  | *p* | HR | lower | | upper | *p* | HR | lower | upper |
| Age at diagnosis (years) | < 60 | 0.095 | 1.000 | |  |  | 0.237 | 1.000 |  |  |
|  | 60-69 | 0.313 | 0.799 | 0.516 | | 1.236 | 0.325 | 0.802 | 0.516 | 1.245 |
|  | 70-79 | 0.194 | 0.746 | 0.480 | | 1.160 | 0.453 | 0.842 | 0.538 | 1.319 |
|  | 80+ | 0.015 | 0.469 | 0.255 | | 0.862 | 0.044 | 0.529 | 0.284 | 0.984 |
| Histological type | SCC |  | 1.000 |  | |  |  | 1.000 |  |  |
|  | Adenocarcinoma | <0.001 | 2.616 | 1.639 | | 4.174 | <0.001 | 2.560 | 1.587 | 4.129 |
| Tumor size (T) | T1 | 0.006 | 1.000 |  | |  | 0.001 | 1.000 |  |  |
|  | T2 | 0.134 | 1.366 | 0.908 | | 2.054 | 0.315 | 1.248 | 0.810 | 1.921 |
|  | T3 | 0.917 | 1.035 | 0.539 | | 1.989 | 0.674 | 1.159 | 0.584 | 2.300 |
|  | T4 | <0.001 | 2.303 | 1.431 | | 3.706 | <0.001 | 2.844 | 1.671 | 4.840 |
| Nodal status (N) | N0 |  | 1.000 |  | |  |  | 1.000 |  |  |
|  | N+ | 0.001 | 1.799 | 1.263 | | 2.564 | 0.181 | 1.314 | 0.881 | 1.961 |
| Grading (G) | G1/2 | 0.052 | 1.000 |  | |  | 0.173 | 1.000 |  |  |
|  | G3/4 | 0.027 | 1.494 | 1.047 | | 2.134 | 0.124 | 1.332 | 0.924 | 1.919 |
|  | GX/ns | 0.617 | 0.829 | 0.398 | | 1.728 | 0.469 | 0.759 | 0.360 | 1.600 |
| Lymph vessel invasion (L) | L0 | <0.001 | 1.000 |  | |  | <0.001 | 1.000 |  |  |
|  | L1 | 0.004 | 1.969 | 1.250 | | 3.103 | 0.397 | 1.269 | 0.732 | 2.200 |
|  | LX/ns | 0.238 | 0.784 | 0.523 | | 1.175 | 0.003 | 0.326 | 0.155 | 0.683 |
| Venous invasion (V) | V0 | 0.005 | 1.000 |  | |  | 0.017 | 1.000 |  |  |
|  | V1 | 0.005 | 2.785 | 1.372 | | 5.655 | 0.034 | 2.290 | 1.064 | 4.928 |
|  | VX/ns | 0.505 | 0.885 | 0.617 | | 1.268 | 0.014 | 2.250 | 1.179 | 4.294 |

*p-value in line of reference denotes p-value for entire variable

CI=Confidence interval

HR=Hazard ratio

SCC=Squamous cell carcinoma

ns=non-specified

**Suppl. Table E:** Cumulative distant metastasis recurrence rates according to patient and tumor characteristics (univariable and multivariable Cox regression)

**Supp Fig. A: Cumulative locoregional recurrence rate and distant metastasis recurrence rate**


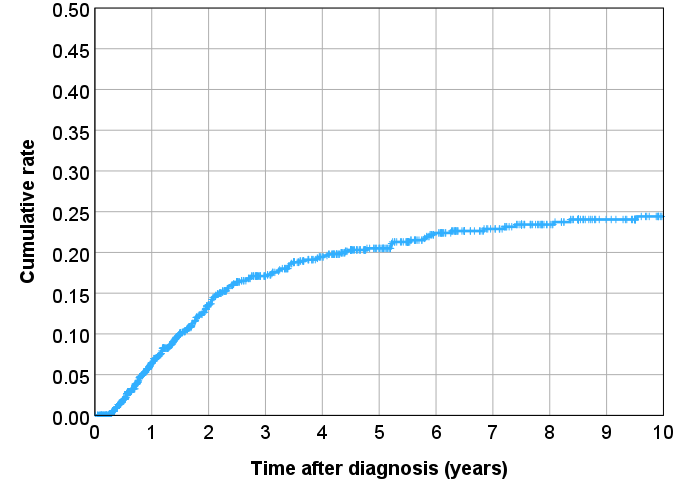


Cumulative locoregional recurrence rate


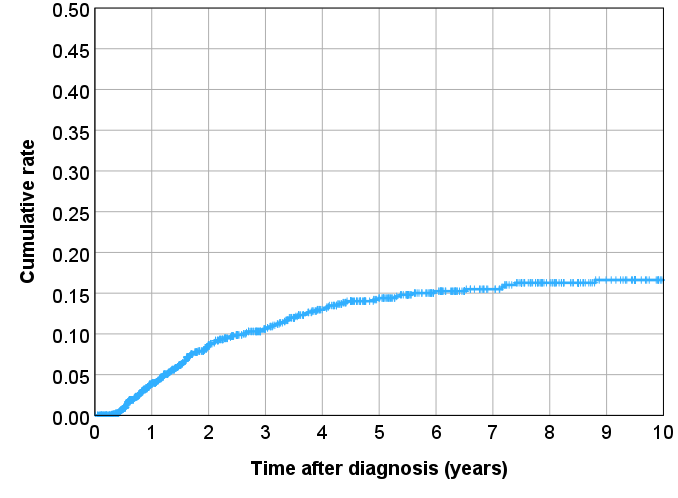


Distant metastasis recurrence rate

**Suppl. Figure A:** Cumulative locoregional recurrence rate and distant metastasis recurrence rate

**Abbreviations**

| **Abbreviations** | **Meaning** |
| --- | --- |
| CI  CT  HR  OP  OP+RCT  OR  OS  RCT  RT  SCC  SD  SEER  TNM  UICC | Confidence interval  Chemotherapy  Hazard ratio  Surgery  Surgery plus radiochemotherapy  Odds ratio  Overall survival  Radiochemotherapy  Radiotherapy  Squamous cell carcinoma  Standard deviation  Surveillance, Epidemiology, and End Results  Tumor, Node, Metastasis  Union international contre le cancer |
